# Pulmonary Embolism Diagnosed During Endobronchial Ultrasound in a Patient With Major Trauma

**DOI:** 10.7759/cureus.35273

**Published:** 2023-02-21

**Authors:** Tetsuro Maeda, Alexander Fe, Sandhya Khurana, Michael Nead

**Affiliations:** 1 Division of Pulmonary and Critical Care Medicine, University of Rochester Medical Center, Rochester, USA

**Keywords:** trauma, computed tomography pulmonary angiography, pulmonary artery, endobronchial ultrasound, pulmonary embolism

## Abstract

Pulmonary embolism (PE) is a serious condition that often poses a diagnostic challenge. We report a case of a 57-year-old man with tobacco dependence who presented with multiple trauma, with chest imaging findings concerning for malignancy. While performing bronchoscopy with endobronchial ultrasound (EBUS), an echogenic material was incidentally found in the left pulmonary artery. Computed tomography pulmonary angiography (CTPA) was immediately obtained and confirmed the diagnosis of PE. This case illustrates the utility of routine pulmonary artery examination during EBUS procedures in patients at risk of PE and the importance of prompt management including confirmation with CTPA.

## Introduction

Pulmonary embolism (PE) is a common and potentially life-threatening disease, which often poses a significant diagnostic challenge. Once diagnosed, prompt interventions such as therapeutic anticoagulation are indicated. While computed tomography pulmonary angiography (CTPA) is considered the standard diagnostic modality for PE [1], endobronchial ultrasound (EBUS) has been shown to have the ability to detect clinically significant thrombus in pulmonary arteries [2]. However, it has not been well studied how to utilize EBUS for the diagnosis of PE, or what would be the best approach for patients incidentally diagnosed with PE during EBUS. We present a case of PE incidentally diagnosed during EBUS performed for a patient with multiple trauma.

This article was presented at the American Thoracic Society 2021 International Conference Thematic Poster Session in May 2021, and its abstract form has been published in the Conference Abstract Issue of the American Journal of Respiratory and Critical Care Medicine (https://doi.org/10.1164/ajrccm-conference.2021.203.1_MeetingAbstracts.A2223).

## Case presentation

**Figure 1:**
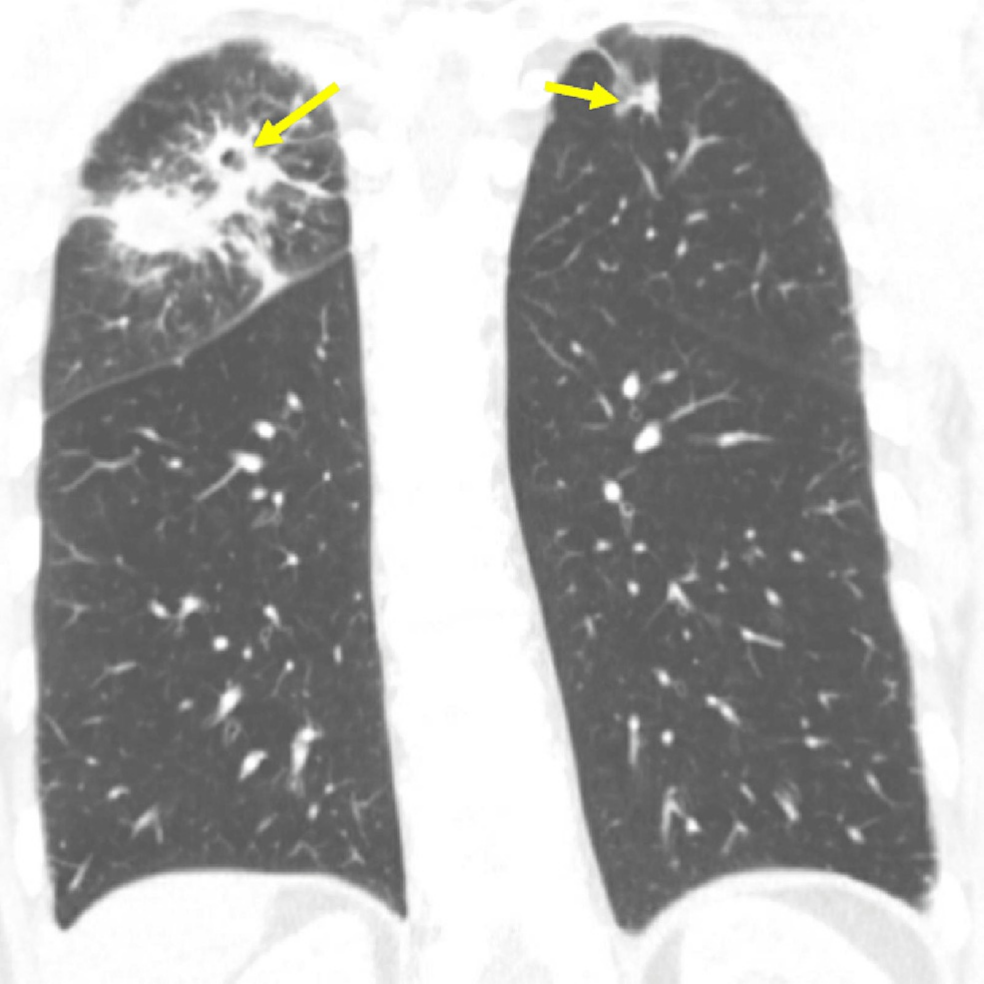
Chest CT showing right upper lobe multinodular patchy consolidation with mild cavitation and left upper lobe nodular consolidation (yellow arrows). CT: computed tomography

A 57-year-old man was admitted to the hospital after a fall resulting in fractures of the right femur, left scaphoid, and multiple lumbar vertebrae. Pulmonary consultation was requested for abnormal findings on chest computed tomography (CT) performed during the initial evaluation for trauma. He reported a two-month history of blood-tinged sputum and weight loss but had no fever, cough, dyspnea, or chest pain symptoms. His past medical history was unremarkable. His social history was notable for current cigarette smoking (49 pack-year) and occupational asbestos exposure while he was in military service, as well as one prior incarceration. The chest CT with contrast showed right upper lobe patchy consolidation with central cavitation and necrosis, left upper lobe nodular consolidation (Figure 1), and mildly enlarged right hilar and paratracheal lymph nodes.

**Figure 2:**
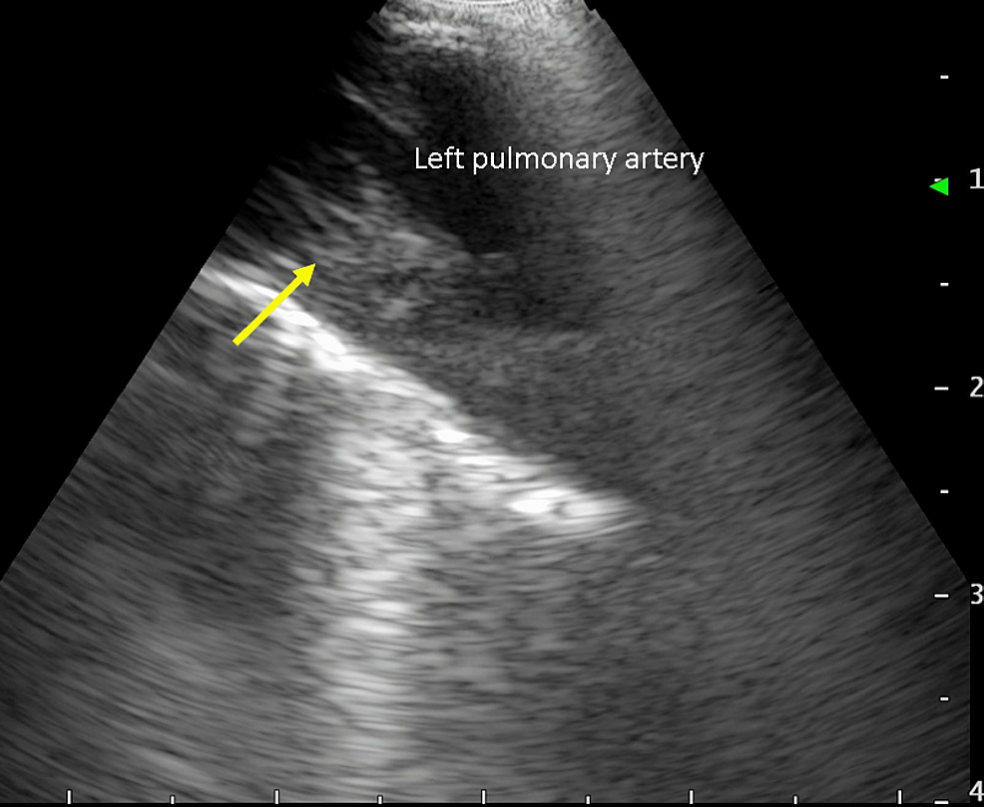
EBUS showing a thrombus in the left pulmonary artery (yellow arrow). EBUS: endobronchial ultrasound

Bronchoscopy was performed with EBUS-guided transbronchial needle aspiration (TBNA) of the hilar and mediastinal lymph nodes, and transbronchial biopsy followed by bronchial brush and bronchoalveolar lavage (BAL) at the right upper lobe lesion. While visualizing the left hilar region with EBUS, the bronchoscopists found an echogenic material in the left pulmonary artery concerning a thrombus (Figure 2).

**Figure 3:**
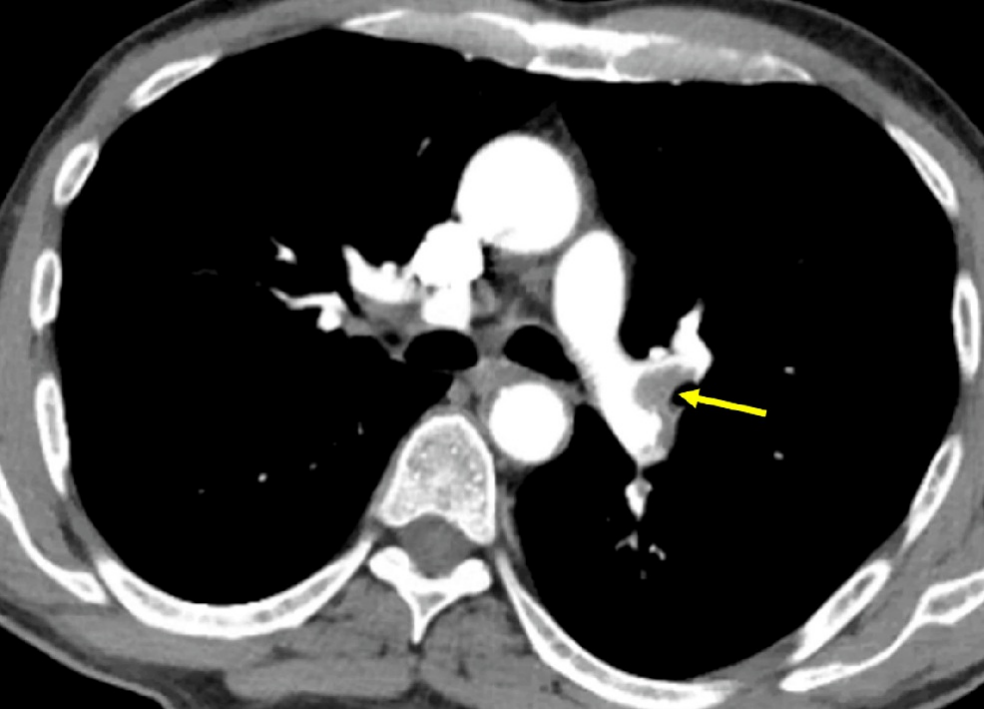
CTPA showing a thrombus in the left main pulmonary artery extending into the left upper and lower lobar branches (yellow arrow). CTPA: computed tomography pulmonary angiography

Consequently, the patient underwent CTPA, confirming new pulmonary emboli in the left main pulmonary artery extending into the left upper and lower lobar branches (Figure 3). While transthoracic echocardiogram showed mildly elevated estimated pulmonary arterial systolic pressure (46 mmHg), right heart size and systolic function were normal and troponin I and N-terminal pro-brain natriuretic peptide levels were normal. Doppler ultrasound of the lower extremities revealed acute deep venous thrombosis in the right popliteal vein. For the venous thromboembolism likely provoked by the trauma, he was started on continuous intravenous infusion of heparin followed by a subcutaneous therapeutic dose of enoxaparin. He remained hemodynamically stable and did not have overt hemoptysis. The anticoagulant was switched to oral apixaban for at least three months upon hospital discharge.

The EBUS-TBNA samples from bilateral paratracheal, subcarinal, and right hilar lymph nodes showed cellular evidence of lymph nodes without malignant cells. A transbronchial lung biopsy of the right upper lobe lesion showed fragments of lung parenchyma with chronic inflammation, interstitial thickening, and reactive pneumocytes. The bronchial brush showed hemosiderin-laden macrophages, and the bronchial brush and BAL fluid specimens were negative for bacteria, mycobacteria, or fungi. While the exact etiology for the abnormal chest CT findings remained unclear, malignancy appeared less likely and therefore a decision was made to manage pulmonary contusion and possible infection. He was given amoxicillin-clavulanate for seven days and a repeat chest CT in six weeks was recommended. Because he resided more than 100 miles away, he elected to follow up with outside medical providers near his home.

## Discussion

To the best of our knowledge, the present case is the first report of PE in a patient with multiple trauma incidentally diagnosed with EBUS. It illustrates two potential implications in clinical practice.

First, although EBUS is primarily used to aid in TBNA, bronchoscopists should consider the examination of the pulmonary vasculature, especially in patients who are at high risk of developing PE. In 2008, Casoni et al. first reported that EBUS is useful in differentiating PE from pulmonary artery sarcoma in a patient with equivocal CT findings [3]. Aumiller et al. then performed a prospective multicenter pilot study on 32 patients with PE diagnosed with CTPA (a total of 101 radiographically distinct thrombi). They performed EBUS within 24 hours of CTPA and demonstrated that EBUS could confirm 97 out of the 101 thrombi detected with the CTPA. Four thrombi not visible with EBUS were all peripherally located and diagnosis of clinically significant PE would have been established with EBUS in all 32 patients. The procedure was feasible with moderate sedation with a mean procedure time of 3-5 minutes [2]. Following this, there have been multiple case reports of PE incidentally diagnosed with EBUS. Underlying conditions included malignancy [4], pulmonary sarcoidosis [5], pulmonary histoplasmosis [6], and pulmonary tuberculosis [7]. Erer et al. found that among 548 patients undergoing EBUS for various reasons, four were incidentally diagnosed with PE [8]. Given the short time required to perform pulmonary artery examination during EBUS, the minimal risk associated with the evaluation, and the clinical implications of making a diagnosis of PE, the careful vascular examination should be part of every EBUS as a complete evaluation to catch incidental findings such as noted in this case.

Second, if an incidental diagnosis of PE was made with EBUS, prompt confirmation with CTPA would be reasonable. Recent case reports describe that EBUS can be used as a primary diagnostic test in critically ill patients who are too unstable to undergo CTPA. This includes a patient in cardiac arrest who improved with intravenous thrombolysis therapy for massive PE diagnosed with bedside EBUS [9], and patients on extracorporeal membrane oxygenation [10]. However, there has yet to be a consensus on the diagnostic accuracy of EBUS for PE and current guidelines for PE do not mention EBUS as a diagnostic modality [1]. Almost all case reports describing PE initially diagnosed with EBUS note that CTPA was later performed to confirm the diagnosis [2,4-8,10]. Therefore, unless contraindicated, confirmation with a better-established diagnostic modality such as CTPA would likely be the best approach.

## Conclusions

We describe a case of PE incidentally diagnosed during EBUS performed for a patient with multiple trauma. Bronchoscopists should consider performing a pulmonary artery examination routinely during EBUS procedures, particularly in patients who are at high risk of developing PE. If EBUS findings are concerning for PE, confirmation with better-studied modalities such as CTPA would be reasonable as long as it can be immediately performed. Future studies are desired to further establish the role of EBUS in the diagnosis of PE.
